# Widespread increase in frequency and duration of European wind droughts based on CMIP6 projections

**DOI:** 10.1016/j.isci.2026.115075

**Published:** 2026-02-18

**Authors:** Idunn Aamnes Mostue, Guillermo Valenzuela-Venegas, David Ruiz Banos, Trude Storelvmo, Marianne Zeyringer

**Affiliations:** 1 Department of Technology Systems, University of Oslo, P.O. Box 70, Kjeller, 2027 Oslo, Norway; 2 Department of Mathematics, University of Oslo, P.O. Box 1053, Blindern, 0316 Oslo, Norway; 3 Department of Geosciences, University of Oslo, P.O. Box 1047, Blindern, 0316 Oslo, Norway

**Keywords:** atmosphere modeling, environmental management, environmental science

## Abstract

To combat climate change, wind power represents an important energy source. However, the availability and reliability of wind resources are subject to weather conditions and the potential impact of climate change. This study utilizes five CMIP6 model projections under the high-emission SSP5-8.5 scenario to analyze future wind speeds and extreme wind events across Europe for the periods 2040–2059 and 2080–2099. We find a widespread increase in the annual frequency of weak wind events up to 10%–20% in some regions, most significantly over western Europe. While winter frequencies for weak winds may decrease in some regions, the duration of these events is projected to rise in northern and eastern Europe. Conversely, and with less model agreement, strong wind events could increase by up to 70% in the southern North Sea and offshore northern Spain. These results underscore the critical need for climate adaptive and climate-resilient energy systems planning.

## Highlights

• Climate change impacts both low and high extreme wind speed events
• Increase in future annual low-wind events over western Europe
• Longer and more frequent strong wind events for certain offshore regions
• Extreme winds need climate-adaptive energy strategies for long-term system stability

## Graphical abstract

**Figure:**
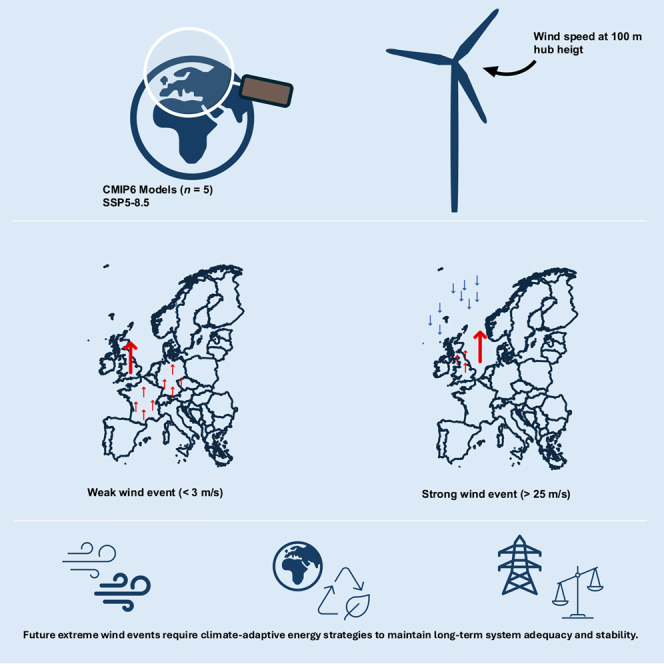

## Introduction

There is an urgent need for long-term sustainable energy solutions, decarbonizing the energy system if we are to reach the Paris Agreement goal of limiting global warming to 2°C and to mitigate the worst consequences of climate change.^1^ Renewable power technologies, which harvest energy from natural resources such as wind and solar radiation, offer a zero-carbon electricity generation solution and thus play a key role in decarbonizing power generation.^2^ As part of the vision of becoming the first climate-neutral continent by 2050, maximizing the deployment of renewable energy and electricity production to meet the energy demand is set as one of the main strategic building blocks by the European Commission to fully decarbonize Europe’s energy supply.^3^ To accelerate the transition to a net-zero energy system, numerous countries have established energy targets to integrate renewable sources into their national energy mix.^4^^,^^5^ However, the transition to renewable-based infrastructure, including wind power generation, presents challenges, particularly concerning the availability of renewable energy sources due to weather conditions and uncertainties in a future changing climate.^6^^,^^7^^,^^8^

One significant challenge involves the increased frequency and severity of extreme weather events due to climate change.^9^^,^^10^ Such events can compromise the operations of power systems,^11^ leading to the shutdown of renewable energy technologies during adverse conditions.^12^ From a wind energy production perspective, events of wind speed outside the thresholds for power generation (i.e., too weak or too strong wind speeds for power generation) would be characterized as extreme events. To ensure the design of climate-resilient future renewable energy systems, it is imperative to understand the impacts of future extreme weather events on renewable generation.

A number of previous studies analyze the impact of extreme weather events on wind power production in Europe, yet many of them rely on historical data (e.g., Allen et al.^2^; Otero et al.^9^; Raynaud et al.^13^; Antonini et al.^14^; Ohlendorf et al.^15^; Rapella et al.^16^; Bloomfield et al.^17^; and Van Der Most et al.^18^). Various reanalysis datasets are commonly used; Ohlendorft and Schili^15^ use the 50-meter wind speed data from MERRA-2 at 3–6 hourly time resolution running from 1980 to 2019, to study low-wind power events and their frequency and duration over Germany. Antonini et al.^14^ and Rapella et al.^16^ both use hourly 100-meter wind speed data from the European Center for Medium-Range Weather Forecast reanalysis v5 (ERA5). Antonini et al. conducted a historical analysis, running from 1940 to the present, on the global distribution and trends in wind droughts. Rapella et al.^16^ studied large-scale weather regimes related to extreme winds and their impact on offshore wind energy availability over the European panorama from 1950 to 2020. Raynaud et al.^13^ use a selection of datasets, including ERA5 and the European Climate Assessment & Dataset (ECAD), in studying the wind-, solar-, and hydro-power droughts and electricity demand over Europe from 1983 to 2012. Bloomfield et al.^17^ created a European country-aggregated dataset on electricity demand-, wind-, and solar-power generation based on ERA5 and used this for identifying meteorological conditions during peak demand events and the contribution of renewable energy production during these events from 1979 to 2018. Otero et al.^9^ utilize wind power generation from the same dataset as Bloomfield et al.^17^ to analyze the duration and severity of energy droughts during events of low renewable energy generation and high demand from 1979 to 2019. Van der Most et al.^18^ computed electricity production and demand based on ERA5 and present-day capacity distribution, for selected European countries from 2015 to 2021, to construct a framework to examine extreme impact events on the European power system.

Allen et al., Raynaud et al., Antonini et al., and Rapaella et al. define droughts as periods where energy production^2^^,^^13^^,^^14^^,^^15^ or energy supply^2^ falls below a certain threshold. Allen et al.^2^ find that wind droughts over southern Europe are shorter and less intense compared to northern Europe, though still impactful due to average lower wind resources. Similarly, Raynaud et al.^13^ find that over southern Europe, wind droughts are less frequent, but can coincide with solar droughts in winter compared to northern and western Europe. Diversely, Antonini et al.^14^ find longer and more frequent wind drought events over southern and central Europe (e.g., Spain’s interior), compared to the northern Sea coastal areas and the UK, where they find few and shorter wind drought events. Ohlendorf et al.^15^ find more persistent low-wind conditions over Germany in summer and fewer long-duration low-wind conditions over winter. Rapella et al.^16^ find an increase in frequency of high-wind events over the northern Sea and Baltic regions, and a decrease in low-wind events over parts of the North Sea, but an increase in the southern offshore regions of Europe. Otero et al.^9^ find northern and central Europe to be more vulnerable to low-wind events, especially during winter, whereas for southern Europe, wind droughts are less correlated to solar energy droughts. Bloomfield et al.^17^ find peak demand events to be characterized by high atmospheric pressure over Russia and Scandinavia, resulting in very cold temperatures and average-to-low wind speed across Europe, limiting wind power support during critical stress events. They also find that a growth in renewable generation has the potential to reduce peak demand; however, with spatially diverging impact, where Spain could reduce their peak demand to a much larger extent compared to central Europe. While the above-mentioned studies have provided valuable insight into extreme weather events and their influence on, specifically, wind energy generation, they relied solely on historical data, neglecting the potential impact of future climate change.

More recent works in the literature show attempts to address the shortcomings of using historical data only by incorporating future climate projections into their studies, such as the Coupled Model intercomparison Project phase 5 (CMIP5) and CMIP phase 6 (CMIP6; e.g., Carvalho et al.^19^; Martinez and Iglesias^8^; and Esnaola et al.^20^), the Coordinated Downscaling Experiment (CORDEX; e.g., Kapica et al.^21^ use wind capacity factors based on CORDEX climate variables), and single climate model data (e.g., Kay et al.^22^ and Gentile et al.^23^). Carvalho et al.,^19^ Esnaola et al.,^20^ and Martinez and Iglesias^8^ study the wind speed change and corresponding wind energy resources. Carvalho et al.^19^ find a strong decline (10%–30%) across the majority of Europe by the end of the century for the SSP8.5 high-emission scenario. For the same scenario, Martinez and Iglesias^8^ show that for the larger parts of Europe, the wind power density decreases by ∼15%, whereas for large areas of central Europe, there is a negligible change, and a growing wind power density over southern Finland of ∼30% by the end of the century. Kapica et al.^21^ and Kay et al.^22^ look at the future changes in energy droughts, where Kapica et al.^21^ find that the frequency of wind drought days is expected to increase across central Europe in future autumn-spring periods. Moreover, Kay et al.,^22^ defining wind droughts as week-long periods when the 7-day mean wind speed drops below the 20th percentile of historical values, find that there is a >60% chance per winter of experiencing at least one wind drought week over the North Sea. Gentile et al.^23^ find that Europe, especially northwestern and the British Isles, may experience an increase in the frequency and intensity of extreme wind events due to the poleward shift of midlatitude cyclones. Most of the above-mentioned studies have considered low temporal resolution (i.e., daily, weekly, and monthly) wind speed data, limiting the detailed understanding of the impact of extreme weather events on the electricity production over shorter time intervals. Furthermore, a shared drawback in these studies is the reliance on wind speeds at 10 m for analyzing the wind speeds directly at this height or extrapolating to hub height, which may not accurately reflect the conditions experienced at the hub heights of wind turbines and introduce errors in wind potential estimations when values are extrapolated.^24^

Hahmann et al.^24^ propose a new approach to estimating wind speeds to hub height by calculating the height of the sigma pressure levels in a given climate model and further computing the wind speeds to a desired height above ground by interpolating between the levels. They also use higher temporal resolution climate data outputs (i.e., 6-hourly) compared to most of the previously mentioned studies. To our knowledge, there is only one other study, Larsén et al.,^25^ investigating the impact of extreme winds over northern Europe using this model-level data approach and 6-hourly climate data outputs. Larsén et al.^25^ studied the possible effects of climate change on the extreme winds (i.e., 95th-percentile of the wind speed) over northern Europe. Through analysis of 18 CMIP6 models forced with the high emission scenario SSP5-8.5, they find an overall decrease in extreme winds over the Scandinavian Peninsula and most of the Baltic Sea and an increase in the North Sea and the southern Baltic Sea for a near-future period (2020–2049) compared to a historical period (1980–2009).

The study of Larsén et al.^25^ focuses solely on northern Europe and examines high wind events in the near future. However, to effectively plan for future wind energy generation, it is crucial to understand both the frequency and duration of high and low wind events. Infrastructure planning is long-term, and decisions regarding wind energy siting impact future repowering efforts and the layout of transmission lines. A comprehensive understanding of both the near and distant future is essential for making informed and long-lasting infrastructure decisions. Additionally, studying the entire continent of Europe enables the design of energy systems that can benefit from spatial and technological diversification. Therefore, we identify a gap in the literature concerning how both high and low wind events will evolve in the near and far future across the whole of Europe.

The present study aims to contribute to closing this gap, providing an enhanced understanding of the impact of future extreme wind events (both high and low) on wind power production until 2100 across Europe. To do so, we analyze the changes in wind speed and extreme wind events (both high and low extremes) over Europe using global climate projections. Specifically, we focus on studying how these changes may impact the future wind capacities of countries that plan for high penetration of wind power in their energy mix. Our analysis incorporates output at high temporal resolution (i.e., 6-hourly) from selected global climate models (GCMs) of the CMIP6 ensemble, as well as a robustness metric across the models. The latter is a feature made possible by the larger number of models included in the present study compared to most previous work (with the exception of Larsén et al.^25^). We adopt a model-level approach from Hahmann et al.^24^ to estimate wind speed at 100 m above the surface to analyze wind speed estimation at turbine hub heights.

By incorporating future climate projections based on several GCMs and integrating improvements in calculating wind speed at turbine hub height, we aim to provide a better understanding of the implications of extreme wind events for renewable energy planning and policy. This research contributes to broadening the knowledge of climate impacts on renewable energy systems, emphasizing the need for adaptive strategies to ensure the long-term resilience of power systems in the face of changing climate conditions.

## Results

### Wind speed over Europe

#### Wind distribution for selected locations

**Figure 1:**
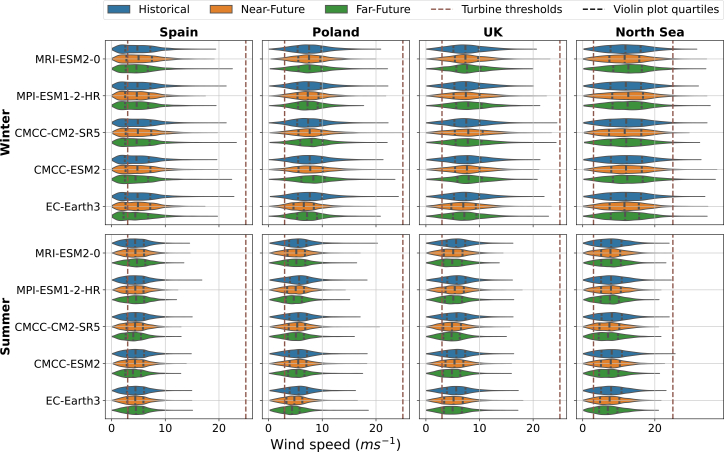
Winter (DJF) and summer (JJA) seasonal wind speed distribution at the grid cell level for four European countries where wind deployment is planned in the next few years Spain, Poland, the UK, and the North Sea. Each violin plot describes the 6-hourly wind distribution of a given climate model and period (i.e., historical [1980–1999], blue; near-future [2040–2059], orange; and far-future [2080–2099], green) for winter (top row) and summer (bottom row). The brown dashed vertical lines indicate the defined operational wind turbine thresholds at 3 ms^−1^ and 25 ms^−1^. The dashed vertical lines within each violin plot indicate the lower, median, and upper quantile (i.e., 25% of the data, median of the data, and 75% of the data).

An overview of the winter (DJF) and summer (JJA) seasonal wind speed distribution at the grid cell level over four locations in Europe (i.e., Spain, Poland, the UK, and the North Sea) is shown in Figure 1, for three time periods (i.e., historical, near-future, and far-future). All four locations were selected based on countries that have planned wind deployment in the next few years.^5^ For these locations, we performed a Kolmogorov-Smirnov (KS) test to evaluate whether the CMIP6 models accurately represent the wind speed distribution, especially its tails that include extreme events, which are the focus of this study. For more details on the methodology used, see the CMIP6 wind speed distribution validation subsection in the method details. The analysis shows that, for extreme-low wind tails, all CMIP6 models accurately reproduce the low-wind speed tail (Figure S9). Conversely, in the comparison of extreme high-wind speed tails, about 60% of the tests successfully replicate the extreme high-wind speed tails, while 40% do not (Figure S10).

**Figure 8:**
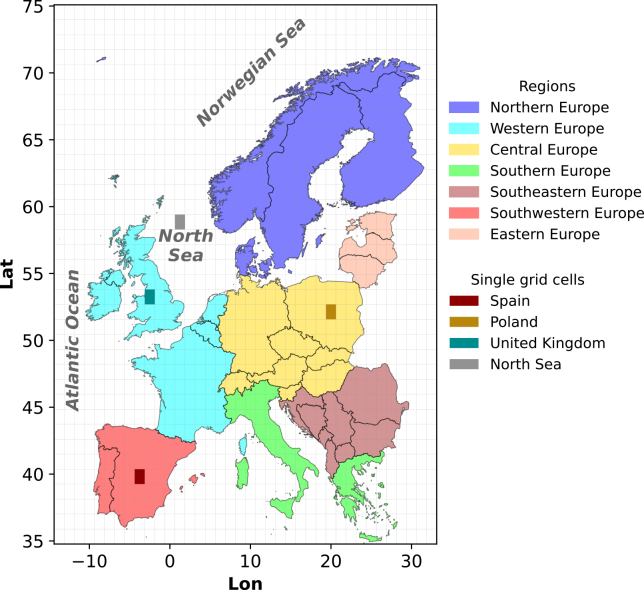
Cutout region over Europe used for the analysis of this study The plot shows the regions considered in the present study and the size of the common grid across the models. The four single grid cells in Spain, Poland, the United Kingdom, and the North Sea, used to compare wind distribution, are also highlighted.

The wind speed distributions at the four locations are skewed to the right, indicating that within each wind speed range, lower wind speeds are more common than higher wind speeds (Figure 1), in line with what we typically would expect following a Weibull distribution (i.e., it is commonly used to represent wind distribution^26^). Moreover, across the four locations, wind speeds below the lower wind turbine operational limit (3 ms^−1^) are seen in the four locations during both the winter and summer seasons, whereas wind speeds above the upper wind turbine operational limit (25 ms^−1^) are only consistently observed in the grid cell over the North Sea in winter. The higher wind speeds in this area can be associated with its offshore location, which generally experiences stronger winds compared to continental areas like Spain and Poland.^27^ Poland’s relatively weak winds could also be related to its proximity to the north Atlantic jet stream exit region.

In the grid cell over Spain, the wind speed distribution shows minimal changes between summer and winter, with average wind speeds around 4–5 ms^−1^ throughout the year. In the grid cell over Poland and the UK, during winter, the average wind speeds across the models range from 6 to 9 ms^−1^, but in summer, they drop to 4–6 ms^−1^. In the grid cell over the North Sea, a more pronounced seasonal difference is observed: while winter wind speeds range from 11 to 13 ms^−1^, the wind speeds drop to 6–8 ms^−1^ in summer.

When comparing the three time periods (i.e., historical, near-future, and far-future), we observe no consistent pattern between every single model nor across the four locations; however, the model spread is larger than the difference between the periods. Moreover, the winter season presents more variability than the summer season for all four grid cell locations. Furthermore, we observe a shorter range in the wind speeds over the continental areas compared to the grid cell over the North Sea.

While the distribution of the wind speed at the four selected locations can give us a pointer to the present-day wind characteristics, the future changes in the wind speed extremes with warming remain uncertain. To further understand the impact of extreme wind events on the future installed capacities and propose a more resilient power system, we need to investigate the changes in wind speeds throughout Europe. In the following section, we study the relative change of future patterns compared to historical data regarding the wind speed mean and extreme wind events over Europe.

#### Wind projection over Europe

**Figure 2:**
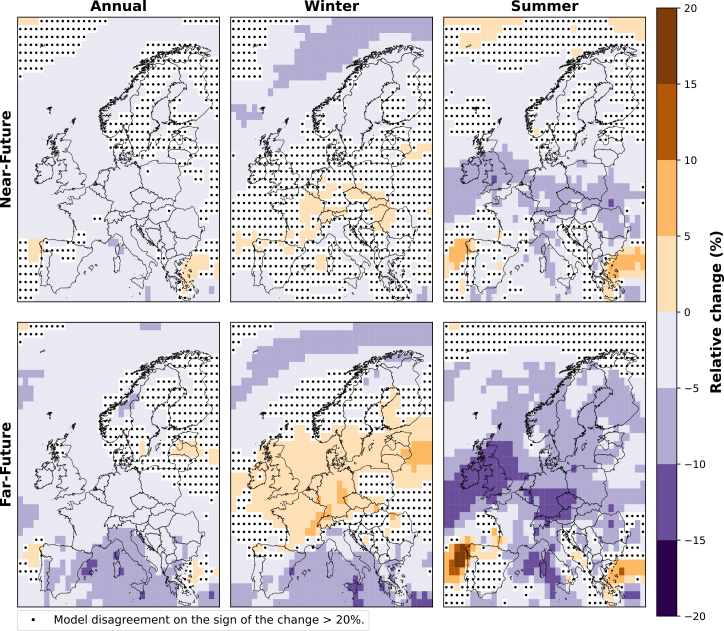
Relative change (%) in wind speed, calculated for the annual, winter (DJF), and summer (JJA) mean wind speeds over the near-future and far-future periods compared to the historical period for the ensemble of CMIP6 models Black dots indicate that two or more models disagree on the sign of the change.

The relative change for the ensemble of CMIP6 models in the annual, winter (DJF), and summer (JJA) wind speeds for the near-future and far-future periods, both relative to the historical period, is illustrated in Figure 2. The model agreement level is indicated with a black dot in each grid box where less than 80% of models agree on the sign of the change (i.e., positive or negative).

An overall decrease in the annual mean wind speed is seen across all regions of Europe, amounting to a reduction of around 5% in both future periods (i.e., near-future and far-future). However, southern Europe shows a more pronounced change in the far-future, exhibiting a decline between 10% and 15% (Figure 2).

In winter, most models project a decline in the wind speed over the Norwegian Sea by approximately 5%–10% for both future periods. However, less model agreement is observed around the coastal region of Norway in the far-future compared to the near-future. Furthermore, parts of western Europe show a 5% increase, while southern Europe shows a 5%–10% decrease.

In summer, we see a reduction in wind speed over most of Europe. The strongest reduction is seen over the UK and nearby offshore areas in western Europe in the far-future, declining approximately 15% compared to the historical period. Conversely, the coastal region of Portugal exhibits the strongest increase in wind speeds of around 15%.

#### Weak wind speed events

Understanding the changes in the frequency and duration of weak wind events across Europe is essential to determine their potential impact on future onshore and offshore wind projects. As follows, we analyze the change in frequency and duration of weak wind events over Europe.

**Figure 3:**
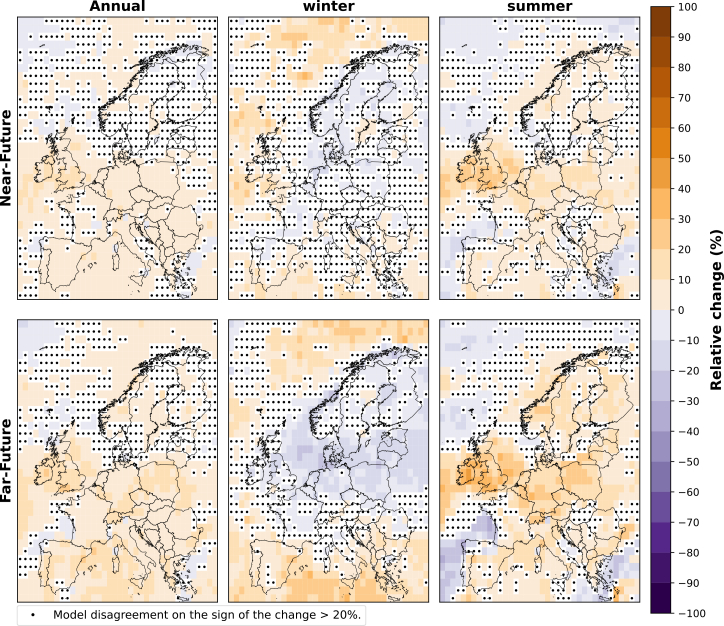
Relative change (%) in the annual, winter (DJF), and summer (JJA) frequency of weak wind events of the near-future and far-future periods compared to the historical period for the ensemble of CMIP6 models The plot shows the median of the relative change over Europe for the model ensemble. Black dots indicate that two or more models disagree on the sign of the change.

Figure 3 presents the relative change for the ensemble of CMIP6 models in the total frequency of future projected weak wind events for the near-future and far-future periods compared to the historical period. We see an increase in the annual frequency of weak wind events over both future periods, with a 10% increase by mid-century and a 10%–20% rise in some regions by the end of the century. The highest increase in the far-future period is observed in western Europe, particularly the UK, with a 20% increase in weak wind events compared to the historical period.

For the far-future winter, most models project a slight decrease in weak wind events in central, eastern, and northern Europe of up to 20% but an increase in offshore areas from 10% to up to 50%. In summer, a 10%–20% increase is projected for the continental regions of Europe by mid-century and even more by the end of the century, reaching 30%–50% over the UK. Conversely, the offshore area west of France, Portugal, and Spain decreases around 10%–20% in the near-future period and up to 40% in the far-future period.

**Figure 4:**
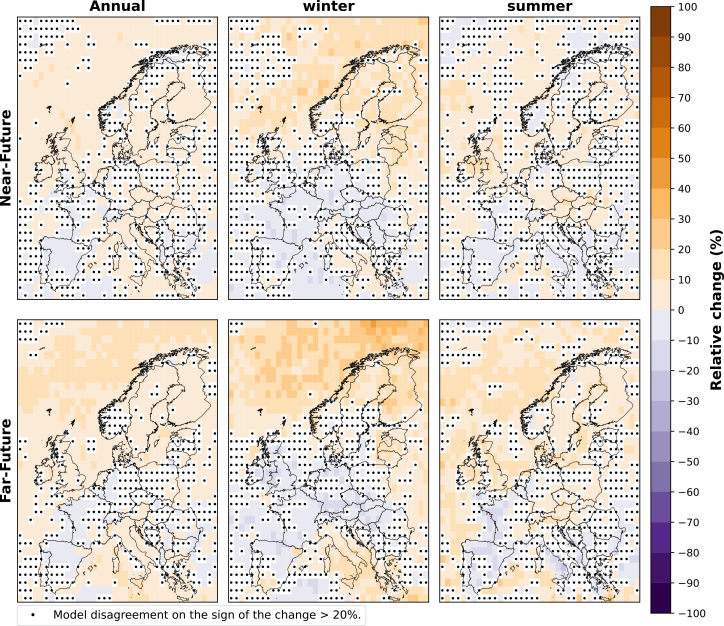
Relative change (%) in the annual, winter (DJF), and summer (JJA) duration of weak wind events of the near-future and far-future periods compared to the historical period for the ensemble of CMIP6 models The plot shows the median of the relative change over Europe for the model ensemble. Black dots indicate that two or more models disagree on the sign of the change.

The relative change in the average duration of weak wind events for the ensemble of CMIP6 models is illustrated in Figure 4. The duration of weak wind events, which typically lasts 12 to 18 h in the historical period (Figure S1 in supplement), is observed to increase overall by up to 10% over most European regions, with a more pronounced rise by the end of the century. In particular, over northern Europe’s offshore area, we observe an increase of up to 20% in the far-future, while only south-western Europe (north of Spain and west of France) experiences slight decreases of less than 10%.

In winter, northern Europe shows a 30% increase in weak wind event duration and up to 40% in specific offshore areas in the north of Norway in both future periods (Figure 4). Southern Europe shows a 10% increase, consistent with annual changes. Conversely, western and central Europe show decreases of up to 10% in the near-future and 20% in the far-future, contrasting the annual trend, which shows increases of around 10% for these areas in both future periods.

In summer, the average duration of weak wind events is projected to increase during both future periods over central and eastern Europe, as well as the UK and Ireland, reaching up to 10%–20% in the near-future and 30% in the far-future. On the other hand, most of south-western Europe will experience decreases, showing reductions of less than 10% in the near-future and up to 20% in the far-future.

#### Strong wind speed events

**Figure 5:**
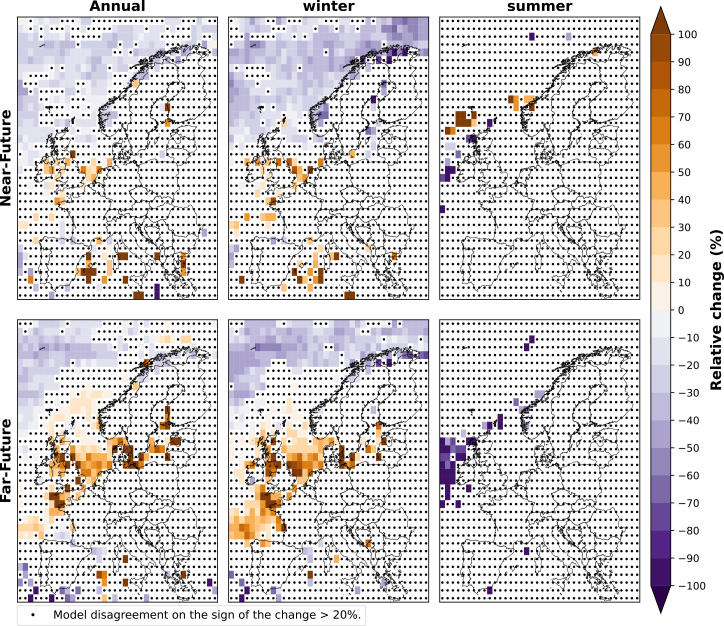
Relative change (%) in the annual, winter (DJF), and summer (JJA) frequency of strong wind speed events during near-future and far-future periods compared to historical period for the ensemble CMIP6 models The plot shows the median of the relative change over Europe for the model ensemble. Black dots indicate that two or more models disagree on the sign of the change.

**Figure 6:**
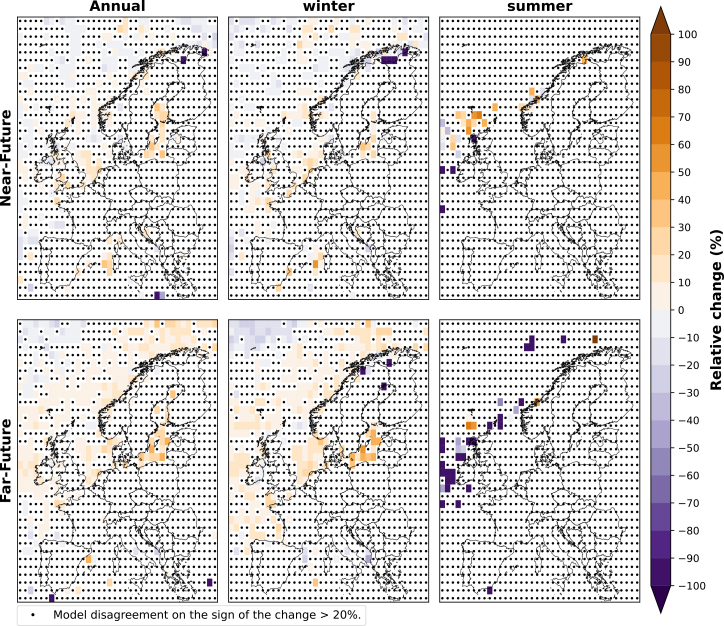
Relative change (%) in the annual, winter (DJF), and summer (JJA) duration of strong wind speed events during near-future and far-future periods compared to historical period for the ensemble of CMIP6 models The plot shows the median of the relative change over Europe for the model ensemble. Black dots indicate that two or more models disagree on the sign of the change.

The relative change for the ensemble of CMIP6 models in the annual, winter (DJF), and summer (JJA) frequency and duration of strong wind events for the near-future and far-future periods, both relative to the historical period, is illustrated in Figures 5 and 6. In contrast to weak wind speed events (Figures 3 and 4), the changes in the strong wind speed events are generally less robust (i.e., the areas in which there is model agreement on the sign of the change are very limited). This could partly be explained by the fact that extremely high wind speeds (i.e., above 25 ms^−1^) are hardly ever reached over continental areas, especially (Figure 1).

We observe a decrease in the projection of the annual frequency of strong wind events for both future periods over the Nordic Sea and the Atlantic Ocean southwest of the UK and Ireland (Figure 5). Moreover, while the relative change shows up to 40% decrease in the near-future period, the Bay of Biscay and the southern North Sea show an increase of up to 70% in the frequency of strong wind events at the end of the century compared to our historical reference.

As for the annual change in strong wind events, a similar pattern is observed in winter (Figure 5). The near-future period shows a decrease in strong wind event frequencies across the Norwegian Sea and over the Atlantic Ocean north-west of the UK and Ireland of around 40% over most of these areas. Diversely, in the far-future period, the offshore areas in the northern part of the Norwegian Sea, northern Spain, and the southern part of the North Sea project an increase in the frequency of strong wind events of up to 70%–90%. In summer, we observe no pattern in the frequency of strong wind events for both future periods. This is due to the almost nonexistence of high extreme wind speeds (above 25 ms^−1^, as seen in Figure 1), so there is little model agreement, as there is little to no data to compare.

Although the duration of strong wind speed events generally shows less model agreement compared to weak wind events (Figure 6), the far-future period shows consistent agreement among the models throughout the year, especially in winter, with projections indicating an increase in the duration of strong wind events in the offshore areas of northern and western Europe, which typically last 6–12 h in the Historical period (Figure S1 in Supplement). These regions (i.e., northern and western Europe) also showed an increase in the duration of weak wind events (Figure 4), suggesting longer-lasting extremes at both ends in the future. This change is observed throughout the periods and is particularly notable in winter, with a relative increase of up to approximately 20%. However, in winter, some areas, specifically over the sea in the northwest of the considered area, show a projected decrease in the duration of strong wind events by up to 30% by the end of the century. Similarly, as for the frequency of strong wind events, we observe no pattern in the frequency of strong wind events for both future periods in summer.

#### Overall changes in extreme weather events

In the previous sections, we have examined how weak and strong wind events are projected to change in the near-future (mid-century) and far-future (end of the century) under the high-emission future scenario SSP5-8.5. The models suggest that weak wind events will become more frequent and last longer across most of Europe, with annual frequency and duration increasing by up to 20%, aligning with the studies mentioned above (Allen et al., Raynaud et al., Kapic et al., Kay et al., and Gentil et al.), where the authors highlight that over central, northern, and western Europe, the frequency and intensity of wind droughts are expected to rise. However, in winter, the models indicate a decrease in the annual frequency of weak wind events by 20%–30% in northern, central, and eastern Europe. The average duration of these events during winter is expected to decrease by up to 20% only in specific areas of south-western, western, and central Europe.

In contrast, the models project a general decrease in the annual frequency of strong wind events by 20%–40%, except over the North Sea and offshore areas in the north of Spain and west of France, where the frequency could increase by up to 40% or higher in some grid cells. These findings are in line with Larsén et al.,^25^ where the authors find a decrease in high extreme wind speed over the Scandinavian peninsula during the near future (2020–2049) but an increase in high extreme wind events over the North Sea. This implies that over the Norwegian Sea, strong wind events will become less frequent but last longer, while in the North Sea and south-western offshore areas, these events will be both more frequent and longer.

Comparing these findings with the projected changes in mean wind speed (Figure 2), we find a general trend showing a decrease in mean wind speed across most of Europe, except during winter, where central, western, and eastern regions show an increase in wind speed at the end of the century. This shift impacts extreme wind events, leading to an increase in both the frequency and duration of weak wind events, as seen in Figure 3. In contrast, strong wind events reduce in frequency over the north of Europe but increase in duration. This pattern is different over the North Sea and offshore areas in western Europe, where, although wind speeds are projected to decrease, the strong wind events are projected to increase in frequency and duration.

#### Implications for power system planning

Power system planning studies usually use historical data to design future energy systems.^12^ As a consequence, if the renewable generation changes due to climate change, the system’s resilience could be compromised, potentially leading to a failure to meet electricity demand. Therefore, it is important to analyze future projections, particularly regarding extreme wind events that could directly affect the operation of wind plants.

**Figure 7:**
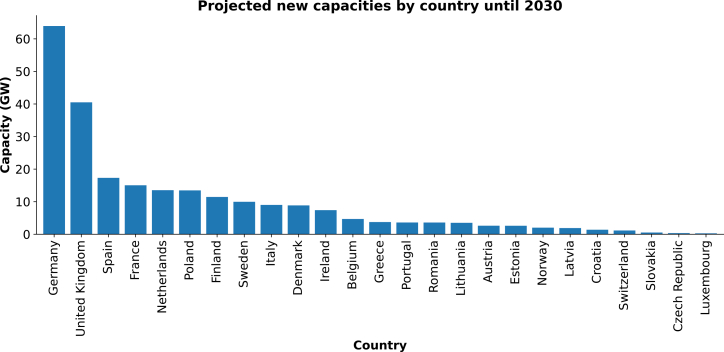
Future wind capacities (GW) projected from 2024 to 2030 by European countries according to WindEurope Each bar shows the total wind capacity projected to be installed between 2024 and 2030 per country.

In the previous section, we used wind speed projections from CMIP6 to analyze their changes in extreme wind events, contrasting their trends across Europe. However, the future implications for power system planning remain uncertain, largely dependent on the specific locations of new wind energy deployments as well as the seasonal pattern of power consumption and generation. Figure 7 illustrates future wind projects across various European countries.^5^ The countries with the highest projected capacities include Germany, the UK, Spain, France, the Netherlands, Poland, Finland, Sweden, Italy, and Denmark. Particularly, Germany is expected to lead with nearly 64 GW of additional capacity installed over the next six years, while the other countries follow with an average future planned capacity of 15 GW.

Table 1 presents the projected changes by the end of the century (far-future period) in both the annual frequency and average duration of weak wind events across various countries. The data presented in this table have been calculated using the annual frequencies and average duration at the grid cell level from Figures 3 and 4, and subsequently weighted according to the area within each country to consider its borders. In winter, nearly all countries with significant wind power deployment are projected to experience a reduction in the frequency of weak wind events. Conversely, during summer and throughout the year, these countries show an increase in the number of weak wind events. Regarding the average duration of weak wind events, an increase is projected for northern European countries such as Sweden, Denmark, and Finland during winter. In contrast, the countries located in western and central Europe are projected to experience a decrease in duration during this season. Throughout the summer and the year, most of these countries are projected to experience a lengthening in the duration of weak wind events. On the other hand, Spain is an exception to these general trends. Here, we observe more frequent but shorter extreme wind events throughout the year and in both seasons, except for winter, where the events are projected to last longer. Moreover, Italy will, according to the model results, experience more frequent and longer weak wind events during the year and both seasons (Table 1).

**Table 1:** Relative change in the annual frequency and average duration of weak wind events during the far-future period for the ten countries with the highest projected wind capacities until 2030

Countries	Frequency (%)			Duration (%)		
	Winter	Summer	Annual	Winter	Summer	Annual
Germany	−5.32	15.41	8	−4.46	7.49	3.13
United Kingdom	−6.91	19.21	11.23	−5.92	10.6	5.33
Spain	6.48	5.56	7.6	3.09	−11.3	−3.14
France	−0.83	9.2	6.42	−3.93	−1.25	−0.86
Netherlands	−14.48	16.89	7.24	−3.44	1.95	2.27
Poland	−10.55	12.66	9.33	−3.61	7.18	1.84
Finland	−6.48	12.19	6.93	11.36	4.22	3.55
Sweden	−2.49	11.59	7.27	6.07	4.88	3.24
Italy	4.31	6.11	7.52	4.24	4.12	4.31
Denmark	−14.81	5.92	1.61	1.69	10.7	5.69

From a European energy system perspective, winter is a critical season to study because electricity consumption significantly increases due to weather conditions^28^^,^^29^: low temperatures and reduced daylight hours. Additionally, solar power generation is limited during this season, making energy systems more reliant on other sources like wind and hydroelectric power. This reliance is evident in the higher electricity prices observed in winter, particularly in the Nordic countries.^30^

Scenarios where the frequency and/or duration of weak wind events increase during winter are particularly challenging for energy systems with a high share of wind generation envisaged. For example, as seen from this study, both the frequency and duration of weak wind events are projected to increase over Spain and Italy in winter. This change could lead to overestimating future energy generation capacities due to increased intermittency in wind plant operations. A long-term seasonal energy storage system^31^^,^^32^^,^^33^ could be a viable solution to address this issue. This system would store energy generated during periods when wind generation is less affected, such as in the summer, leveraging other renewable sources like solar power.

In some northern European countries, such as Finland and Sweden, the frequency of weak wind events is, in this study, projected to decline during winter. This reduction in operation intermittency will allow these countries to produce more electricity than at historical levels. The higher output (compared to historical) could compensate for the longer duration of weak wind events. Alternatively, these countries could implement short-term storage systems to store surplus energy generated due to the projected reduction in weak wind events.

In western and central European countries, such as the Netherlands, the UK, France, and Germany, both the frequency and duration of weak wind events are projected to decrease. This scenario should not negatively impact the resilience of their energy systems, as the reduced intermittency will result in more stable wind plant operations.

## Discussion

In the present study, we assess the implications of changing wind speed and extreme wind events when planning future wind energy projects across Europe until 2100. We aim to provide insights into the potential impacts and guide decision-making processes and infrastructure planning to ensure the resilience of power systems in the face of climate change and the transition to renewable energy sources. To achieve this goal, we analyze future climate projections based on GCMs from the CMIP6 ensemble. By adopting a model-level approach, we estimate wind speed at turbine hub heights, enhancing the reliability of these calculations.

Our findings reveal that weak wind events are expected to increase in frequency and last longer throughout the year, except in winter, when their frequency is expected to decrease. These results suggest that while power systems need to enhance their flexibility by incorporating alternative renewable energy sources and energy storage systems (e.g., batteries) throughout the year, the impact on power supply during the winter season, when electricity demand increases due to household heating, is not predominant. In contrast, and with less model agreement than for weak wind events, strong wind events are projected to increase in duration but decrease in frequency. This can be interpreted as good news for the wind energy sector as fewer exceedances of the cut-out wind speed means fewer generation breaks and, hence, more generation.

In terms of regional impacts, we see a 10%–20% rise in the annual frequency of weak wind events in certain regions by the end of the century, with notable increases in the United Kingdom. The increase in weak wind events we observe over northern Europe (Figure 5) could relate to the poleward shift of the North Atlantic storm track and associated midlatitude westerlies, particularly in winter, leading to reduced cyclone frequency over European midlatitudes seen in CMIP6 projections.^34^ Strong wind events increase by up to 70% in the offshore regions of northern Spain and the southern North Sea during the far-future period.

The changes in the frequency and duration of weak and strong wind events pose significant challenges for optimizing energy generation and maintaining power system stability, highlighting the necessity for resilient power system planning considering the changing wind resource availability across different regions. However, the main challenge in studying these implications is the availability of high-resolution weather variables in GCMs, which limits the representativeness of the intrinsic uncertainty across models in the future projections. Even though we provide an assessment, there remains a significant gap between the available data provided by GCMs and the electricity sector for achieving a robust evaluation.

As countries strive to achieve their renewable energy targets, it becomes increasingly important to address the implications of climate change on the reliability and resilience of power systems. By acknowledging and proactively responding to the potential shifts in wind speed patterns and extreme weather events, policymakers can effectively integrate wind energy into existing power grids and foster a more sustainable and resilient energy future. Climate-resilient system design could include expanding transmission infrastructure, allowing to take advantage of spatial diversity in wind generation long and short-term storage as well as optimizing the turbine design for the location and its potential change in wind energy output.

### Limitations of the study

This study examines changes in wind speed distribution across Europe and their potential impact on wind turbine operations for the mid-century (2040–2059) and the end-century (2080–2099) periods. To achieve this, we use high temporal (i.e., 6-hourly) wind speed data derived from GCMs available in CMIP6. The study acknowledges several limitations, some of which are listed below. In future studies, further addressing these limitations could provide a better understanding of how wind speed distribution changes might impact wind turbine operations across Europe in the future.

• Global climate model ensemble and temporal resolution: the selection of GCMs used for the present analysis is limited by the availability of climate variables with high temporal resolution (i.e., 6-hourly). Accurate estimation of extreme events in a given area requires data with finer temporal and spatial resolution. However, fulfilling this requirement reduces the number of available models, thus reducing the models available to validate the results. Moreover, we assumed the 6-hourly wind speeds remained constant throughout each time step, even though they could vary significantly within this time frame of 6 h. This assumption was necessary to calculate the duration and frequency of extreme events. Future studies could use temporal downscaling or higher-resolution data to capture temporal fluctuations more accurately.
• Availability of GCMs: a key limitation in this study is the reliance on a relatively small ensemble of five GCMs, compared to other studies that use more than 15 models (e.g., Hahmann et al.^24^ and Larsén et al.^25^). In this work, the total number of models was limited by the need for high-temporal-resolution weather variables across multiple vertical pressure levels, reducing the available GCMs from 28 to just six, with one later becoming unavailable for download. These requirements were necessary for accurately interpolating wind speed to the wind turbine hub height of 100 m. However, this limitation in GCM availability has two main implications: representativeness and challenges in using these data for electricity system models. Firstly, the small ensemble may not fully represent the entire CMIP6 dataset for future scenarios, which could lead to under- or overestimation of the uncertainty related to projected extreme wind events. The relatively small ensemble size influences the balance of the uncertainties in our results. We find that the spread between the models is substantially larger than the difference between the two future periods (i.e., near-future and far-future, Figure 1). This pattern is consistent with Hawkins and Sutton (2009),^35^ who showed that model uncertainty is the dominant source of projection spread for mid-to late 21st century regional climate projections. While the current results offer a preliminary assessment, using a larger and more diverse set of GCMs would improve the robustness of extreme weather projections. Secondly, the absence of more GCMs meeting the specific requirements presents a significant challenge for their use in electricity system modeling, as precise wind speed data are crucial. Analyses based on limited GCMs may carry uncertainties that can propagate through the modeling process. Additionally, as wind turbine technology advances toward higher hub heights—exceeding 100 m, for example—the need for data across multiple vertical levels will grow more urgent. Relying on 10-m wind speeds or extrapolating over large vertical distances could lead to significant inaccuracies. Future efforts should focus on providing high-resolution vertical data to improve the accuracy and reliability of climate change impact assessments on the electricity sector.
• Spatial resolution: the CMIP6 data used in this study contain the instantaneous values of climate variables at a grid resolution of approximately 100 x 100 km. This coarse resolution results in an oversimplification of features such as over mountainous areas, where wind speed can vary a lot, and cannot capture smaller scale features, e.g., katabatic winds and sea breezes.^36^ Additionally, many wind farms are situated in coastal regions, where climate models, due to their coarse resolution, will struggle to correctly assign land vs. sea. While the present approach may overestimate or underestimate extreme wind speed values due to the extension of each grid cell, it provides a general understanding of future trends in extreme wind events over Europe and under the defined high-emission scenario SSP5-8.5. Future research could benefit from spatial downscaling using higher-resolution datasets like CORDEX.
• Operational wind turbine limits: we defined weak and strong wind events using the most common operational thresholds for wind turbines^27^: 3 ms^−1^ for the lower boundary and 25 ms^−1^ for the upper boundary. These thresholds are standard but can be adjusted depending on turbine specifications and local operational decisions. We based our decision on the most common values for our analysis. Future research might include sensitivity analyses of these thresholds to understand their impact on future extreme wind event trends, especially relevant for offshore wind with new designs and higher cut-off values, as is the case of the wind turbine V236–15.0 MW, which has a cut-off wind speed of 31 ms^−1^.
• Wind speed at 100 m: we interpolated the available wind speed data to approximately 100 m, as this is one of the most common hub height for wind turbines.^27^ Although the choice of hub height can depend on specific wind conditions, we standardize to 100 m to facilitate consistent comparisons across various locations. Future studies could consider using different hub heights tailored to specific onshore and offshore areas for more precise analyses.
• Robustness metric: in this study, five models were employed to analyze the overall trend of wind and strong wind event frequency and duration across Europe over two future periods compared to historical model data. Additionally, we evaluate the robustness of the observed changes across these models, establishing a threshold of 80% model agreement regarding the direction of change. Although this robustness metric^10^ is a helpful practice for determining agreement among model projections, it is not necessarily the most effective method for capturing the variability and uncertainties associated with extreme wind events and their potential impacts on electricity systems. For instance, Figures S2–S5 illustrate the individual relative changes projected by each GCM for weak and strong wind events relative to historical data. Generally, models that disagree with the 80% agreement do not exhibit extreme deviations compared to models that agree. For example, during the far-future period for annual frequency over Nordic countries in summer, 80% of the models project approximately a 20% increase in weak wind event frequency; however, MRI-ESM2-0 shows a roughly 10% decrease in the same period. Conversely, there are only a few locations where extreme disagreement is observed. An example can be seen in the strong wind events, where the general trend aligns with the observed for weak wind events, except in the near-future for their annual frequency during the whole period and winter. In this case, MPI-ESM2-0 exhibits significant disagreement over the Norwegian Sea, showing more than a 100% increase relative to historical data, while the other models project a decrease of about 40%. These discrepancies are crucial to consider from an energy system perspective, as they reflect the diverse possibilities that must be considered in designing resilient electricity systems. Future research should aim to develop improved methods for assessing agreement across models and to investigate the extent of differences among models that do not agree with the majority.

## Resource availability

### Lead contact

Requests for further information and resources should be directed to and will be fulfilled by the lead contact, Idunn Aamnes Mostue (idunnam@uio.no).

### Materials availability

This study did not generate new unique reagents.

### Data and code availability

• All data used to calculate the extreme wind events are listed in the key resources table.
• All of the code used to download the Global Climate Model data and calculate the extreme wind events that support the findings of this study are available at Zenodo: https://doi.org/10.5281/zenodo.18392113.
• Any additional information required to reanalyze the data reported in this article is available from the lead contact upon request.

## Acknowledgments

This work has received funding from the European Union’s research and innovation program 10.13039/100018693Horizon Europe under the agreement no. 101083460, and from the 2022 KD Sustainability grant related to the Faculty of Mathematics and Natural Sciences at the University of Oslo, Norway. We thank the two anonymous referees for their time and insightful feedback.

## Author contributions

Conceptualization, I.A.M., G.V.-V., D.R.B., T.S., and M.Z. ;methodology, I.A.M. and G.V.-V.; investigation, I.A.M. and G.V.-V.; writing – original draft, I.A.M. and G.V.-V.; writing – review and editing, I.A.M., G.V.-V., D.B., T.S., and M.Z.; funding acquisition, M.Z., ; supervision, G.V.-V., D.B., T.S., and M.Z.

## Declaration of interests

We declare that M.Z. is a guest editor of the Special Issue entitled “The Climate-Energy Nexus: Assessing linkages between Climate and Energy Systems” and that they were not involved in the peer-review process of this article.

## Declaration of generative AI and AI-assisted technologies in the writing process

During the preparation of this work, the authors used ChatGPT UiO (GPT University of Oslo) in order to help write Python scripts and notebooks. After using this tool or service, the authors reviewed and edited the content as needed and take full responsibility for the content of the publication.

## STAR★Methods

### Key resources table

**Table:** 

REAGENT or RESOURCE	SOURCE	IDENTIFIER
**Deposited data**		

Climate Model Data	ESGF-DKRZ ESGF-IPSL ESGF-LLNL	https://esgf-metagrid.cloud.dkrz.de/search/cmip6-dkrz/ https://esgf-node.ipsl.upmc.fr/search/cmip6-ipsl/ https://aims2.llnl.gov/search/cmip6/
ERA5 Reanalysis data from the Pan-European Climate Database	Copernicus Climate Change Service	https://cds.climate.copernicus.eu/datasets/sis-energy-pecd?tab=overview

**Software and algorithms**		

Python version 3.11.9	Python Software Foundation	https://www.python.org/
Python package for regridding – xESMF: Universal Regridder for Geospatial Data	Jiawei Zhuang and the xESMF development team	https://xesmf.readthedocs.io/en/latest/index.html
Data acquisition and extreme wind event calculation	This paper	Zenodo: https://doi.org/10.5281/zenodo.18392113

### Method details

In the present study, we examine future changes in European wind speed conditions using five Global Climate Model runs from CMIP6. Specifically, we focus on extreme weather events and their impacts on wind power generation through 2100. We calculate the wind speed at 100 meters above the surface to explore the wind speed at a common wind turbine hub height. We then bias-correct the wind speed values using the Quantile Delta Mapping approach and regrid the data to a common grid across the models and, in the next step, calculate the duration and frequency of extreme weather events. Each step is detailed in the following subsections.

#### Wind speed calculation

The primary variable analyzed in the present work is the horizontal wind speed over time, calculated for a specific hub height covering a cutout over Europe and specific grid cells for which we analyze results in more detail (see Figure 8). All climate variables are retrieved from the Earth System Grid Federation (ESGF) archive of CMIP6 outputs.^37^ We calculate hub-height-specific wind speeds for different heights using CMIP6 climate variables at sigma pressure levels with high temporal resolution of 6-hourly output of instantaneous values – corresponding to the *6hrLev* Table ID in the ESGF CMIP6 archive, following the same methodology as presented by Hahmann et al. (2022). We consider this approach of using variables at sigma pressure levels as opposed to other studies utilizing the power law to extrapolate wind speeds at hub height from 10-meters-above-surface wind speeds, as the latter approach has shown to incur an overestimation of the results over the oceans by not considering their specific atmospheric conditions (e.g., use of constant wind shear) and impacting the installed offshore wind capacities and generation.^24^ Through the approach by Hahmann et al. (2022), the wind speed can be interpolated to any height using Equations 1, 2, 3, and 4 and linear interpolation.

The calculated horizontal wind speed (wnds) can be described through Equation 1, where *u* and *v* are the wind components obtained from CMIP6.

(1) $$wnds=\sqrt{u^{2}+v^{2}}$$

Moreover, Equations 2, 3, and 4 describes the thickness between two model layers *z*_*k*+1_ and *z*_*k*_, where ${\underline{T}}_{V}$ is the virtual temperature average between levels *k* and *k*+1, *ϵ* = 0.622, *R*_*d*_ is the gas constant for dry air, *g* is the Earth’s gravitational constant, *p*_*k*_ is the calculated pressure at the model level *k*, *p*_0_, *a*_*k*_, and *b*_*k*_ are the reference pressure and the sigma level coefficients at the level *k* obtained from CMIP6. Further, *p*_*S*_ describes the surface pressure, and *T*_*k*_ and *q*_*k*_ the air temperature and specific humidity at the model level *k*, respectively.

(2) $$\Delta z=z_{k+1}-z_{k}=\frac{R_{d}{\bar{T}}_{V}}{g}\ln\left(\frac{p_{k}}{p_{k+1}}\right)$$

(3) $$p_{k}=a_{k}p_{0}+b_{k}p_{S}$$

(4) TV=12Tk+1qk+1+ϵϵ1+qk+1−Tkqk+ϵϵ1+qk

Thus, we see that the height of each model level can be obtained by integrating Equation 2 from the surface level to the upper limit of the model. Finally, the wind speed can be interpolated to any height using linear interpolation.

#### Data

For the analysis of this study, we retrieve climate variables from historical simulations (i.e., historical) and future projections (i.e., SSP5-8.5) based on five Global Climate Models (GCMs) from CMIP6 and subsequently convert the climate variables into wind speed and height following the methodology described in the previous subsection. We apply the Quantile Delta Mapping (QDM) approach to bias-correct the data for this study. For the analysis of this study, we retrieve climate variables from historical simulations (i.e., historical) and future projections (i.e., SSP5-8.5) based on five Global Climate Models (GCMs) from CMIP6 and subsequently convert the climate variables into wind speed and height following the methodology described in the previous subsection. We use Quantile Delta Mapping (QDM) to correct any systematic distributional biases in wind speed in this study.^38^^,^^39^^,^^40^ ERA5 reanalysis data^41^ and GCM historical data are used to calculate correction factors, which are then applied to the projection data for the Near-Future and Far-Future periods. A detailed explanation of the methodology is available in Cannon et al. (2015)^38^ and Tong et al. (2021).^40^ Figure S7 in the Supplement depicts the spatial distribution of the absolute mean difference between each GCM model and ERA5 from 2015 to 2024, before and after bias correction. Applying QDM reduces the spatial bias across GCMs, with the maximum absolute bias dropping from 8 ms^-1^ to 2 ms^-1^.

To analyze the impact of extreme weather events on the European renewable energy system, we selected five GCMs: *CMCC-CM2-SR5, CMCC-ESM2, EC-Earth3, MPI-ESM1-2-HR, and MRI-ESM2-0*. These models were chosen because they met the following specific set of data requirements:

i. Data must be available for both ‘historical’ and ‘ssp585’ experimental runs.
ii. Specifically, the ‘ssp585’ scenario data must cover the end-of-century period.
iii. A minimum 6-hourly output is required to capture the dynamics of extreme weather events.
iv. The data must include the following variables: eastward wind (*ua*), northward wind (*va*), air temperature (*ta*), specific humidity (*hus*), surface downwelling shortwave flux in air (*rsds*), and surface upwelling shortwave flux in air (*rsus*).
v. The variables *ua, va, ta,* and *hus* must be available at multiple pressure levels (i.e., *6hrLev*).

Even though the focus of the present study is on wind only, we also consider variables related to modelling solar PV (*rsds* and *rsus*) when filtering the models. Given that future electricity systems are expected to have high shares of wind and solar energy, a joint analysis of these resources using consistent climate model datasets should be pursued in future research. Moreover, we use a cutout of the global data, covering the majority of Europe (see Figure 8 for the cutout region).

Regarding the data collection process, the CMIP6 data utilized in this study were retrieved from the distributed data archive developed and operated by the Earth System Grid Federation (ESGF). To ensure complete data access, three different nodes were employed: LLNL (USA), IPSL (France), and DKRZ (Germany), as specific models were exclusively available on individual servers (last accessed: October 2023). The selection process started with an initial search that identified 28 GCMs with multiple sigma-pressure levels. However, only six satisfied all the established criteria. Furthermore, one of these six could not be downloaded due to persistent server errors, resulting in the final set of five GCMs: *CMCC-CM2-SR5*, *CMCC-ESM2*, *EC-Earth3*, *MPI-ESM1-2-HR*, and *MRI-ESM2-0*.

Although the data from the five different GCMs present a common 6-hourly time resolution, they are inconsistent in their spatial grid resolution (see Table S1 in Supplement) due to the different assumptions behind each model. To facilitate comparison and further calculation across the models, we perform a conservative regridding of all climate data to a common grid resolution of 1.25°x1.12°. We select a new common target grid with a longitudinal and latitudinal size that reflects the largest entity in our model ensemble, so that no grid is being downscaled. Implementing this new common resolution may result in losing some spatial wind speed details, but it enables the direct comparison of wind speed and the measurement of robustness across the models.

We investigate the wind speed distribution and the relative change in the wind speeds by comparing future projections to historical simulations for all five GCMs. We select a 20-year reference period (hereafter *Historical* period) from the historical simulations, running from 1980–1999. We also select two future periods, each of 20–years, from the SSP5-8.5 high emission scenario; one period representing a middle-of-century period running from 2040–2059 (hereafter *Near-Future* period), and another representing an end-of-century period running from 2080–2099 (hereafter *Far-Future* period).

For the analysis of the wind speed distribution, we select four locations – three onshore and one offshore (i.e., Spain, Poland, the United Kingdom: the UK, and the North Sea) – in each of which we pick a single grid cell to represent the respective location (see Figure 8).

#### Extreme wind event definition

To characterize the future changes in extreme wind events across Europe, we calculate the duration and frequency of the extreme events using the peak-over-threshold method.^42^ This method is used in extreme value analysis, selecting all the values above (or below) a certain threshold and then studying their duration and frequency. Every value exceeding the threshold corresponds to an extreme value.

We select two thresholds based on the typical operational wind speed range of wind turbines^27^^,^^43^: the cut-in speed at 3 ms^-1^ (i.e., lower operational limit) and the cut-out speed at 25 ms^-1^ (i.e., upper operational limit). Based on these thresholds, we defined two types of extreme wind events: *Weak wind events* and *Strong wind events,* corresponding to events in which the wind speed is under the lower threshold of the cut-in speed and over the upper threshold of the cut-out speed, respectively.

We count the frequency and duration of extreme wind events at the grid cell level. This is done for each model and every defined period (i.e., Historical, Near-Future, and Far-Future) separately.

The CMIP6 climate variables at 6-hourly output of instantaneous values are constant over the area of each grid cell and treated as a constant throughout each time step, which, for this study, implies that a wind speed falling below or exceeding the set thresholds of an extreme wind event has a duration counted as at least six hours. We acknowledge that the coarse spatial resolution of the CMIP6 models (∼100 km) does not capture the spatial details of the real atmosphere with spatial variability within each grid box. This means that the local wind speed at any given location within a grid box could be higher or lower than the grid box’s mean. The framework of this study does not capture the impact of this spatial variability within a grid box. This could be explored in future work, but would require spatially downscaled CMIP6 outputs.

We calculate the mean duration of the events in each grid cell covering the cutout region over Europe and for each time period (i.e., Historical, Near-Future, and Far-Future). Moreover, the annual frequency (i.e., expected frequency) of extreme weather events is calculated by dividing the total number of events occurring within a certain period by the length of the period in years (i.e., 20 years).

### Quantification and statistical analysis

#### Relative change calculation

For parts of the analysis, we calculate the relative change in the future wind speed compared to the historical wind speed. The relative change is calculated for the annual mean as well as the seasonal mean for the winter months (i.e., December, January, February, DJF) and summer months (i.e., June, July, August, JJA) of each future 20-year period (i.e., Near-Future and Far-Future) compared to the Historical period. This calculation is performed separately for each of the five CMIP6 model runs before we select the median value at each grid point from the model ensemble.

We also consider the relative change in frequency and duration of extreme wind events. In each grid cell for every model individually, we count the annual frequency and mean duration of extreme wind events in the Historical, Near-Future, and Far-Future periods. We then calculate the relative change of extreme wind event frequency and duration for the Near-Future and Far-Future periods compared to the Historical period for each CMIP6 model. Finally, in each grid point, we pick the median value of the model ensemble and display these results in the following section.

Additionally, to consider the robustness of the changes across the five climate models, we define a threshold of 80% (4 out of 5 models) model agreement on the direction of the change among the models (i.e., positive or negative sign of the change). This agreement is calculated for every grid cell and displayed along the resulting maps in the following section.

Individual maps for each GCM showing where the wind distribution of the future period is not significantly different from the historical period can be found in Figure S6 from the Supplement.

#### CMIP6 wind speed distribution verification

To demonstrate that the wind speed distribution from the CMIP6 models, especially their tails, are accurately represented, we compare the historical 100 m wind speed distribution from each of the considered model (i.e., CMCC-CM2-SR5, CMCC-ESM2, EC-Earth3, MPI-ESM1-2-HR, and MRI-ESM2-0) against the ERA5 reanalysis dataset across the four considered subregions (i.e., North Sea, Spain, Poland, and the UK). Specifically, we focus this analysis on two seasons: Winter (DJF: December-February), and Summer (JJA: June-August), as these are the central to this study. We follow a statistical approach, using the Kolmogorov-Smirnov (KS) test to compare the full cumulative probability distributions of the CMIP6 models with ERA5, as well as their tails, using the 5% upper and lower percentiles. All comparisons are performed on the same historical period (i.e., 1980–1999) to ensure fair comparison of model bias. All the steps and assumptions considered are detailed as follows.

The Null Hypothesis (H0) for all tests is that the CMIP6 model’s distribution is statistically the same as the ERA5 distribution. A standard significance level of 0.05 is used. The interpretation of the outcomes of the statistical test is summarized below:

• Statistically similar - p-value >= 0.05 (fail to reject H0): There is statistically significant evidence that the CMIP6 model distribution successfully reproduces the characteristics of the ERA5.
• Statistically different - p-value<0.05 (Reject H0): There is not enough evidence to conclude that the CMIP6 model distribution is significantly different from ERA5.

Using this hypothesis, we test the full distribution of every CMIP6 model and its tails against the ERA5 distribution. Figures S8–S10 in the Supplement show the resulting p-values for each model and location for the full distribution KS-test and the tail KS-test, respectively.
