# Supplementary material for: Widespread increase in frequency and duration of European wind droughts based on CMIP6 projections
Source: iScience. 2026 Feb 18;29(3):115075. doi: 10.1016/j.isci.2026.115075 (PMC12972962; doi:10.1016/j.isci.2026.115075)
Supplement: Document S1. Figures S1–S10 and Table S1 [file mmc1.pdf]

iScience, Volume *29*

Supplemental information

Widespread increase in frequency and duration of European wind droughts based on CMIP6 projections

Idunn Aamnes Mostue, Guillermo Valenzuela-Venegas, David Ruiz Banos, Trude Storelvmo, and Marianne Zeyringer

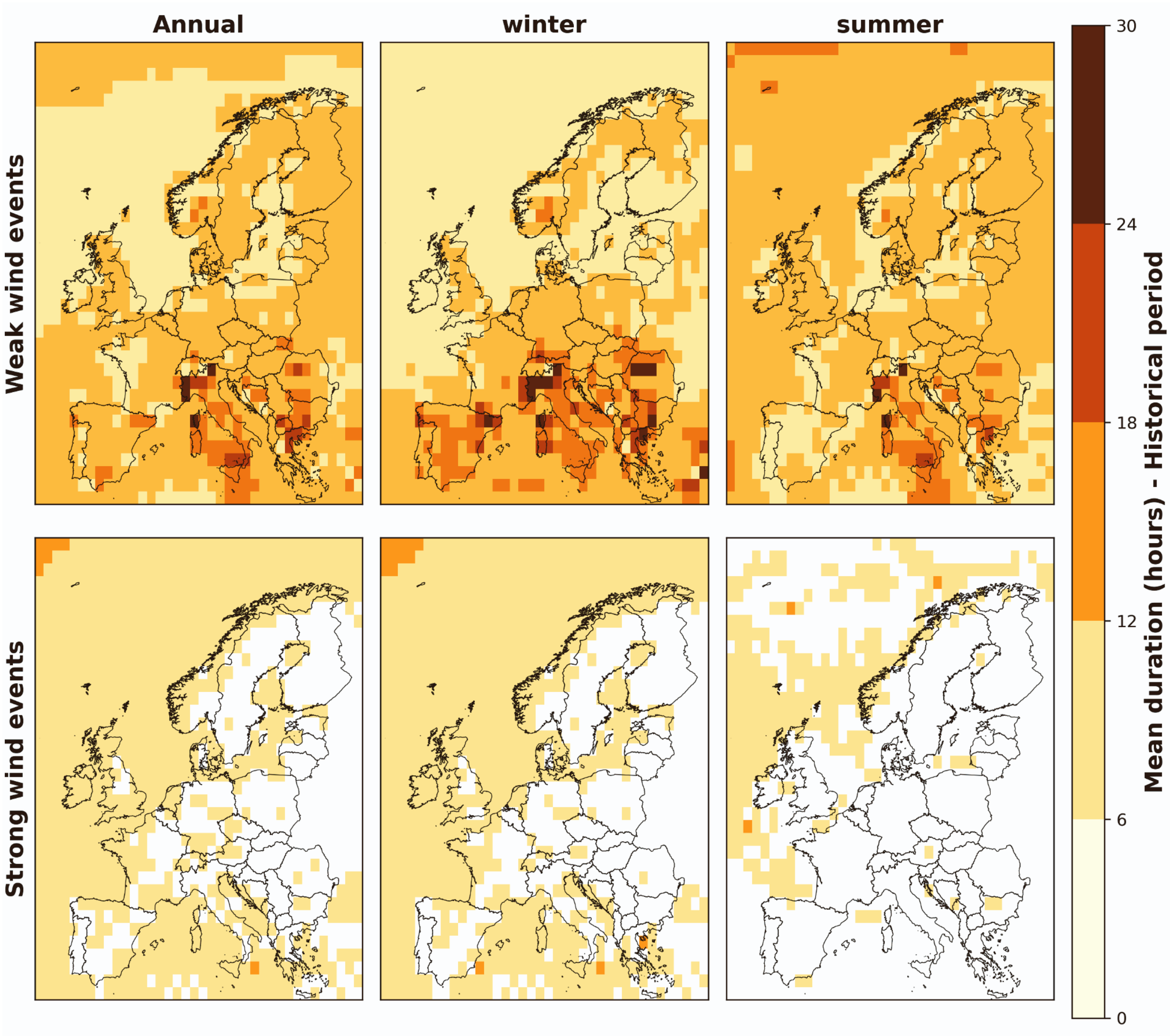

Figure S1: The plot shows the mean duration in the weak and strong wind events over Europe for the model ensemble in the Historical period.

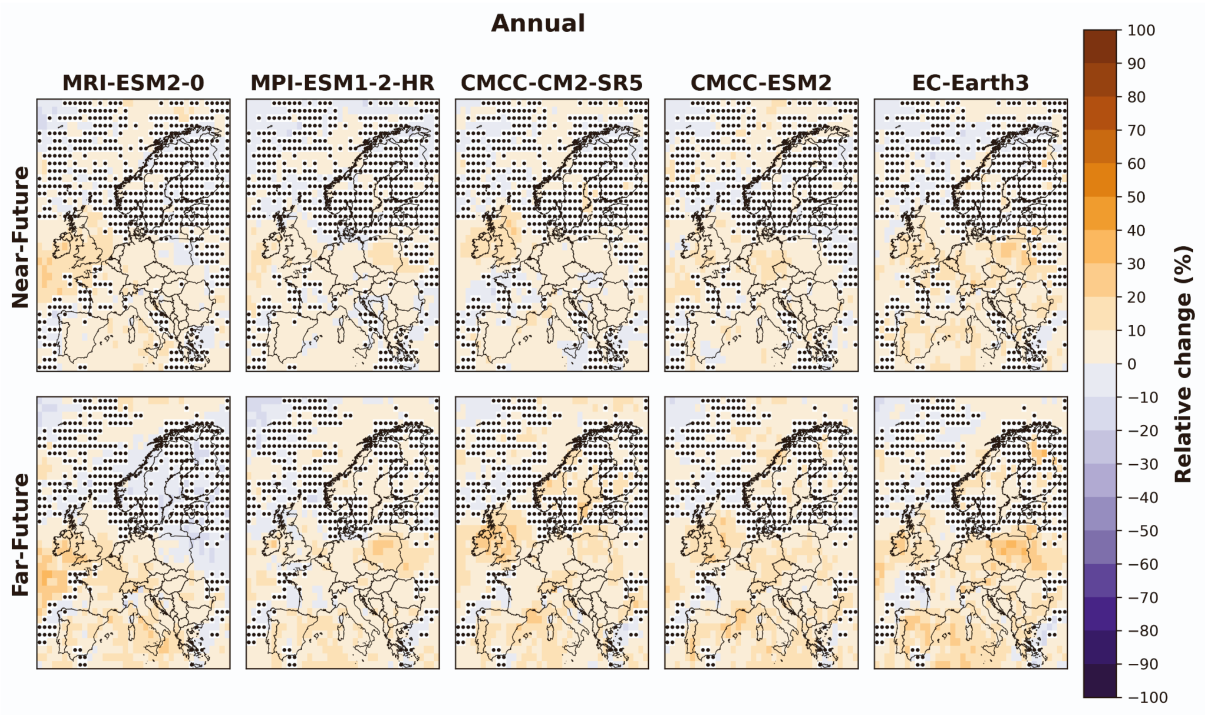

Figure S2: Relative change (%) in the annual frequency of weak wind speed events during Near-Future and Far-Future compared to Historical per selected model. The model agreement of the full model ensemble is added, where black dots indicate that two or more models disagree on the sign of the change.

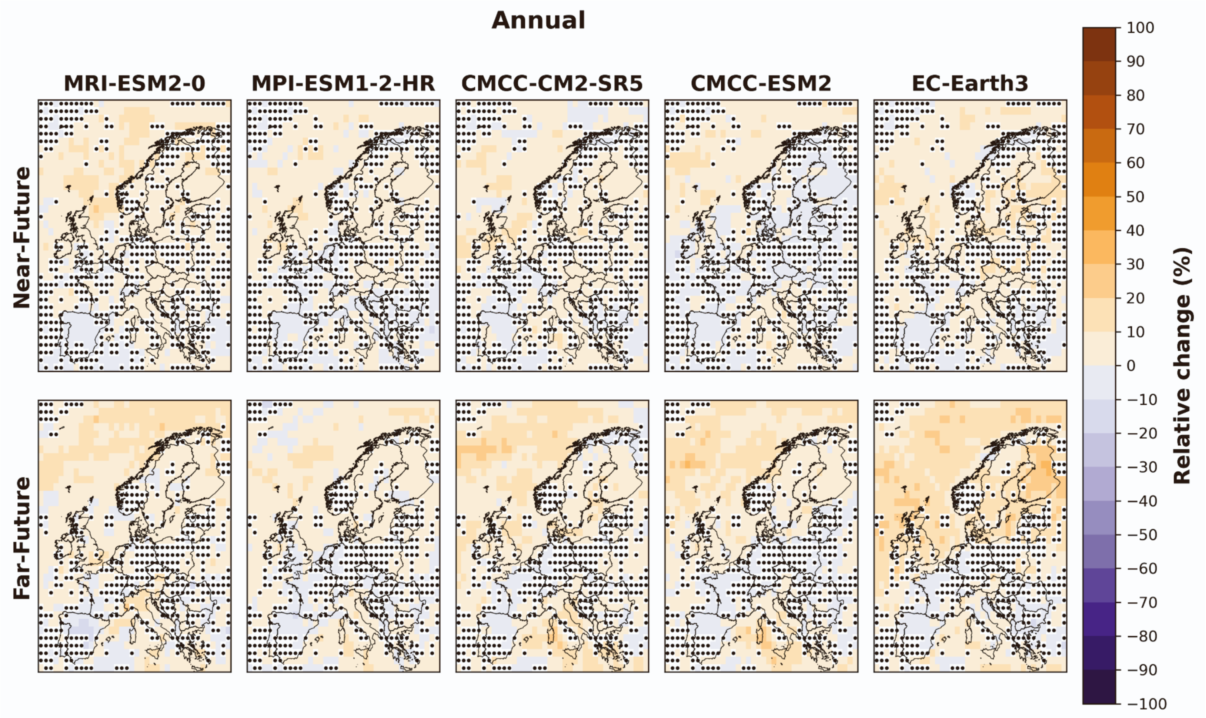

Figure S3: Relative change (%) in the duration of weak wind speed events during Near-Future and Far-Future compared to Historical per selected model. The model agreement of the full model ensemble is added, where black dots indicate that two or more models disagree on the sign of the change.

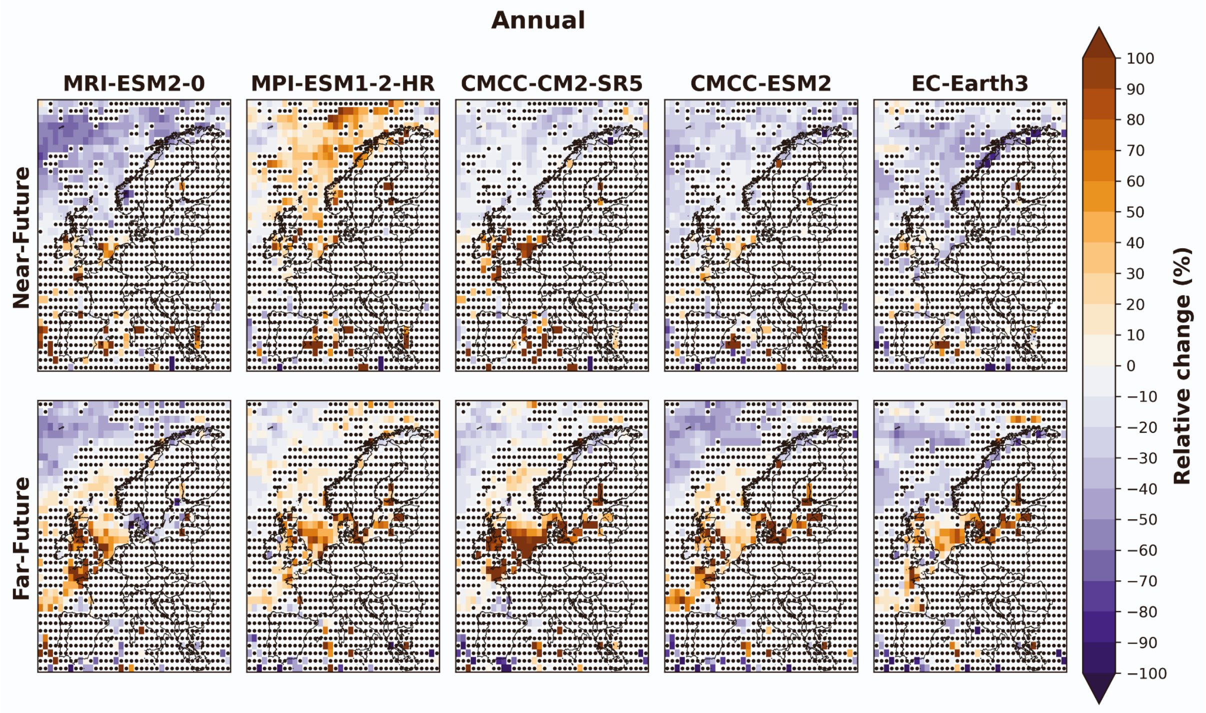

Figure S4: Relative change (%) in the annual frequency of strong wind speed events during Near-Future and Far-Future compared to Historical per selected model. The model agreement of the full model ensemble is added, where black dots indicate that two or more models disagree on the sign of the change.

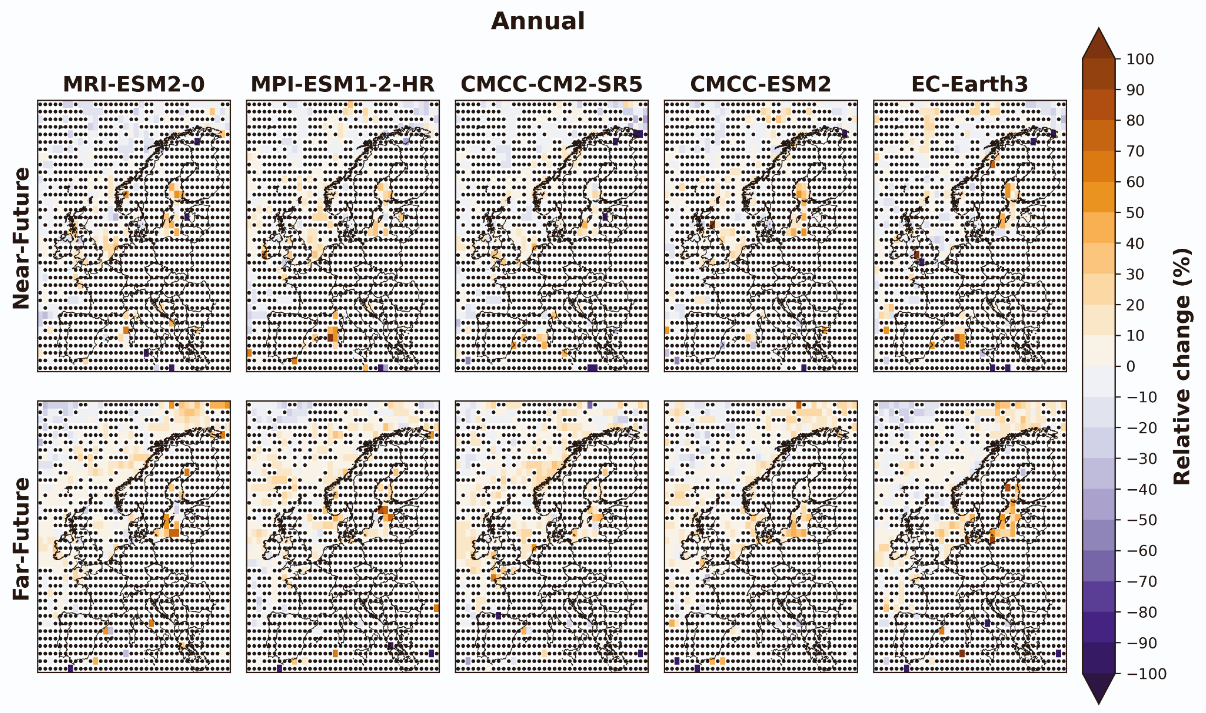

Figure S5: Relative change (%) in the duration of weak wind speed events during Near-Future and Far-Future compared to Historical per selected model. The model

agreement of the full model ensemble is added, where black dots indicate that two or more models disagree on the sign of the change.

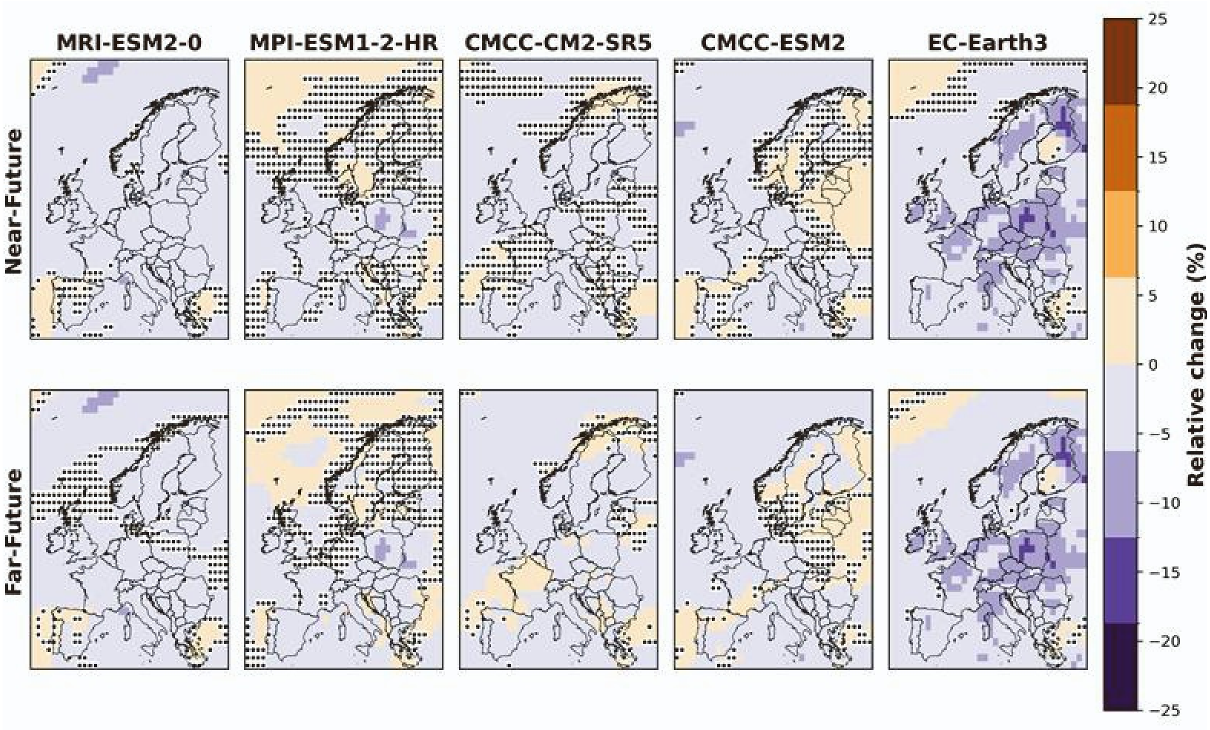

Figure S6: Annual Relative change (%) of wind speed during Near-future and Far-future for each GCM. Black dots indicate areas where the wind distribution of the future period is not significantly different from the historical period.

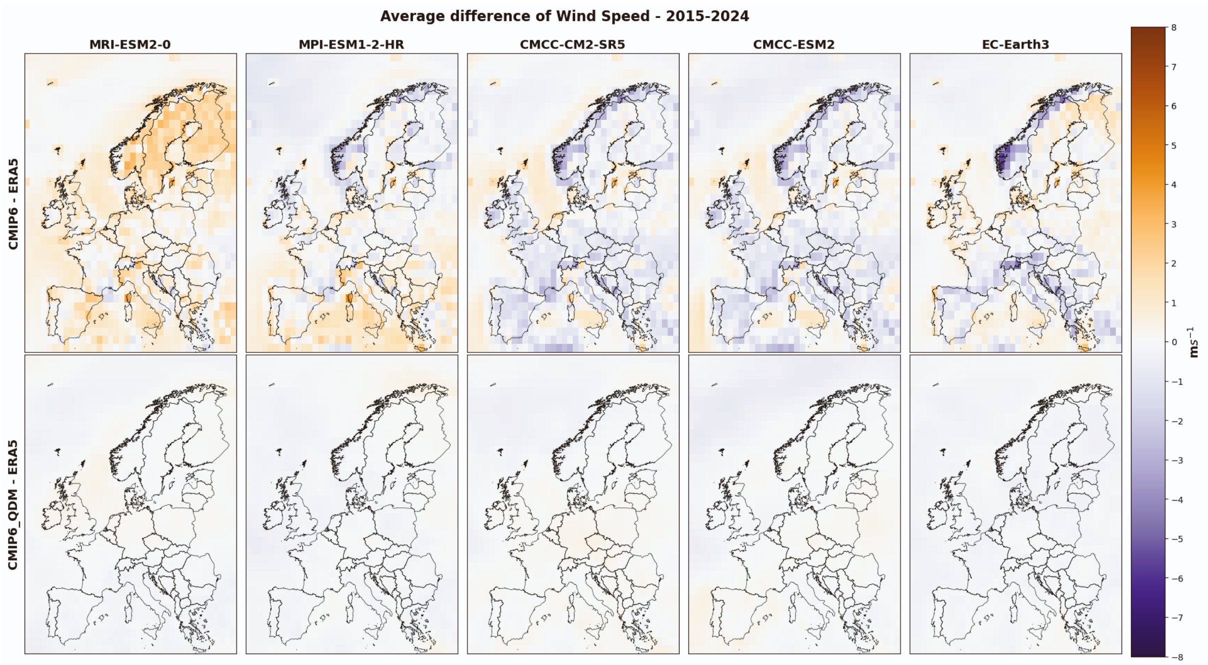

Figure S7: Spatial distribution of the absolute difference (bias) of wind speed (in ms^{-1}) for every GCM model compared to ERA5 data in the period from 2015 to 2024. Each grid cell shows the average bias over the period 2015-2025. The top row shows the bias in the

original GCM data, and the bottom row shows the bias after applying the bias-correction method (QDM).

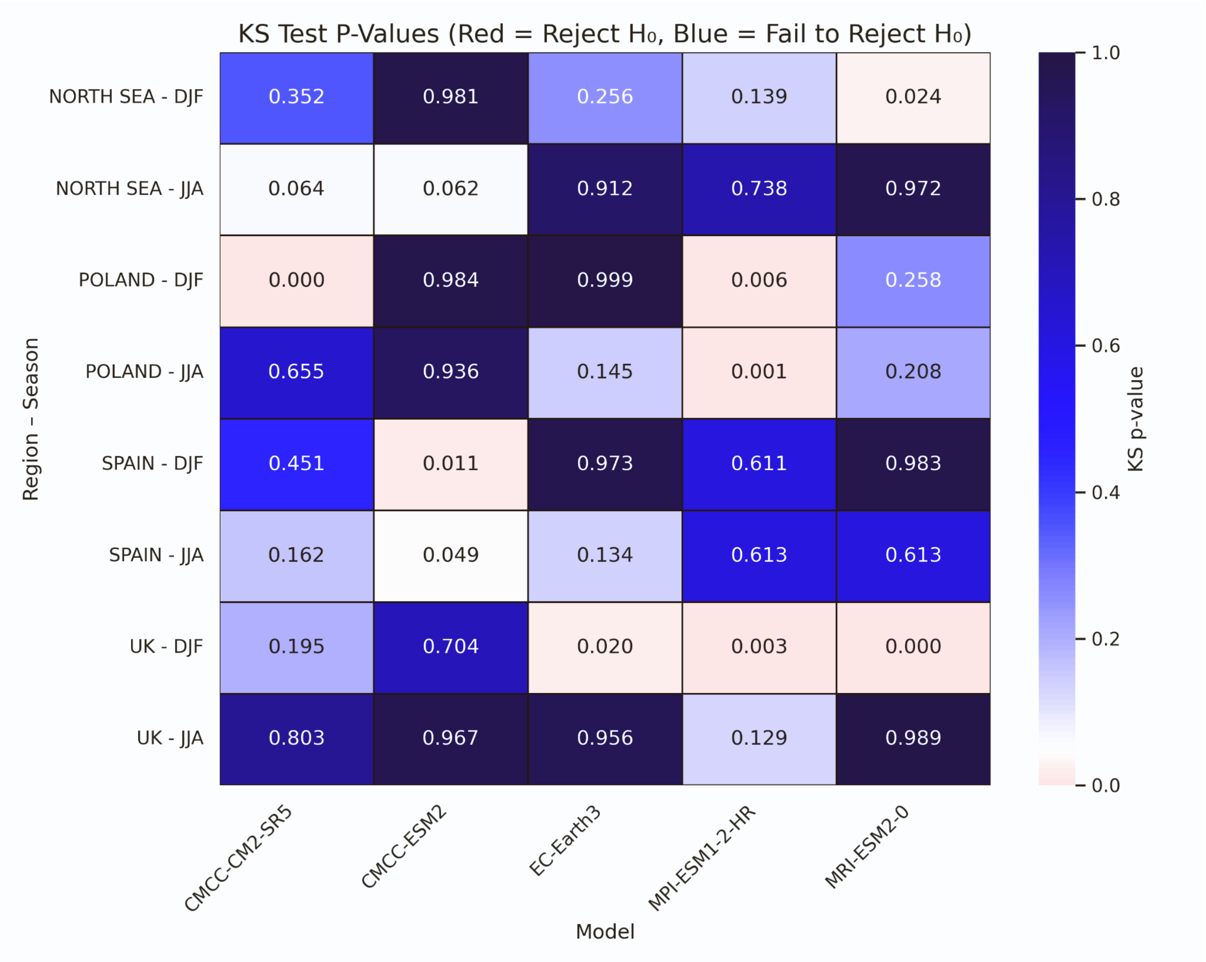

Figure S8: Heat map of p-values from the KS-test for the full distribution of 100 m wind speed. Here we find 80 % of the CMIP6 data to be statistically similar to the ERA5, thus the ensemble of the models generally captures the characteristics of the ERA5 wind speed distribution.

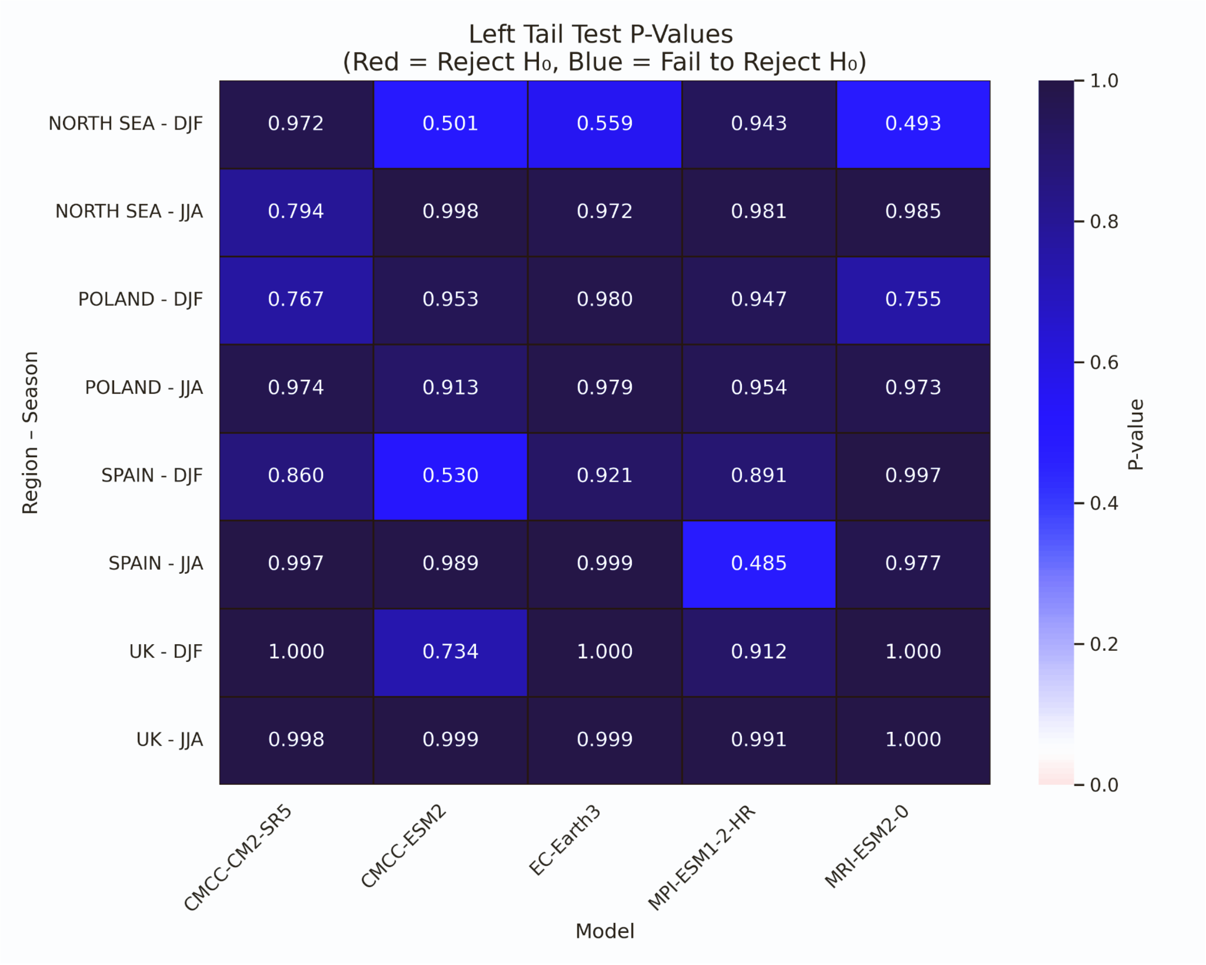

Figure S9: Heat map of p-values from left tail KS-test of the 5th percentile of the 100 m wind speed distribution. Here we find all CMIP6 data to be statistically similar to ERA5, suggesting that the left extreme tail of ERA5 is well captured by the CMIP6 models.

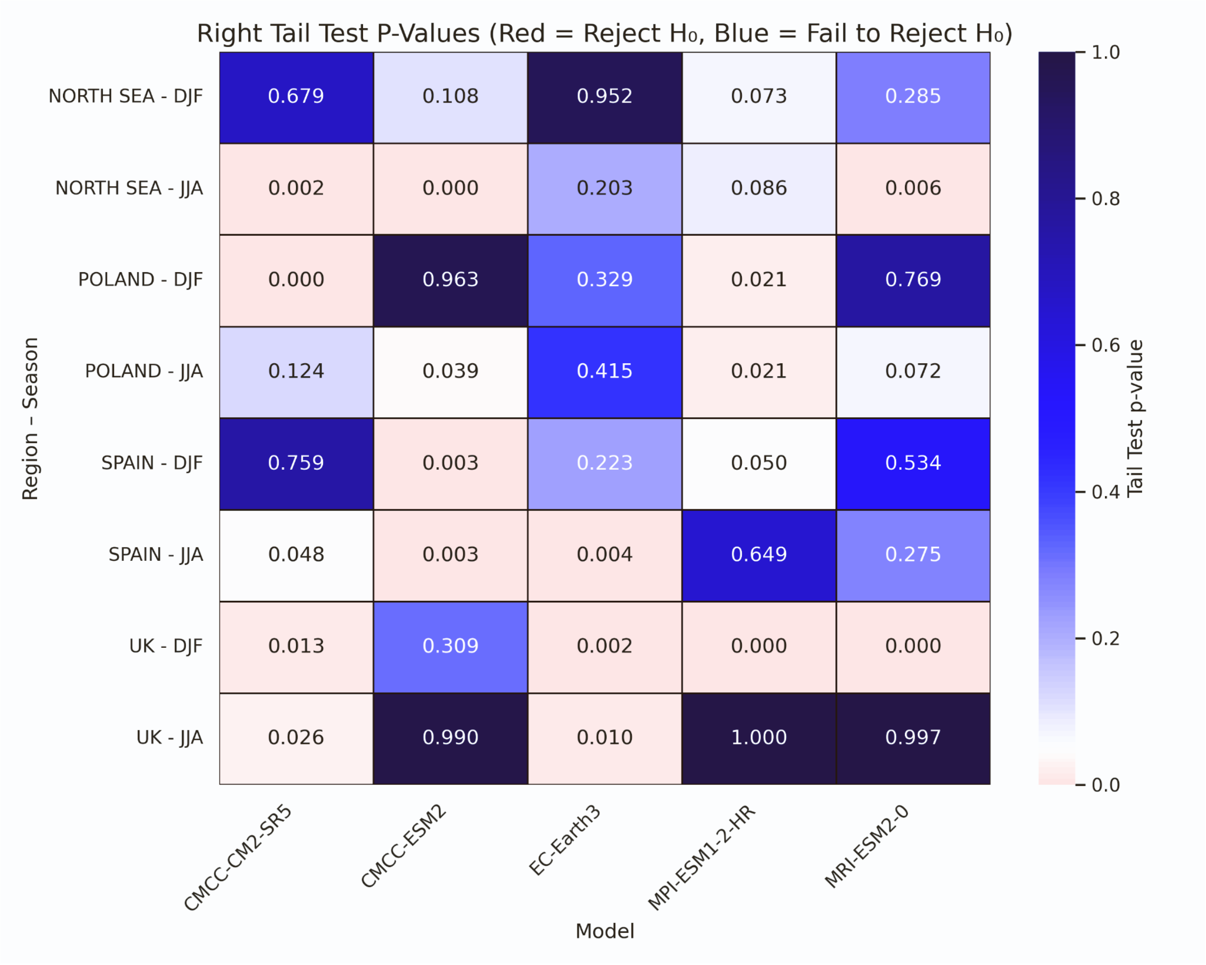

Figure S10: Heat map of p-values from right tail KS-test of the 95th percentile of the 100 m wind speed distribution. Here we find 60% of the CMIP6 data to be statistically similar to ERA5, suggesting that the right tail of ERA5 is not as well represented in the CMIP6 data.

Table S1: GCMs, Institutions, spatial resolution, Calculated heights, Citation, for the CMIP6 models used in this study.

GCM	Modelling Institution – Country	Horizontal grid resolution	Calculated heights below 200 m above ground level	Citation
CMCC-CM2-SR5	Euro-Mediterranean Center on Climate Change (CMCC) Foundation – Italy	1.25°lon x 0.94°lat	60.5, 193.8	Cherchi et al. (2019)[S1]
CMCC-ESM2	Euro-Mediterranean Center on Climate Change (CMCC) Foundation – Italy	1.25°lon x 0.94°lat	60.5, 193.8	Lovato et al. (2022)[S2]
EC-Earth3	EC-Earth Consortium	0.7°lon x 0.7°lat	9.3, 31.7, 62.9, 104.1, 155.3	Döscher et al. (2022)[S3]
MPI-ESM1-2-HR	Max Planck *Institute* for Meteorology Earth System *Model* – Germany	0.94°lon x 0.94°lat	30.6, 138.9	Müller et al. (2018)[S4]
MRI-ESM2-0	The new Meteorological Research *Institute* Earth System *Model* – Japan	1.12°lon x 1.12°lat	11.8, 35.5, 67.2, 115.0, 183.0	Kawai et al. (2019)[S5]

Supplemental references

S1. Cherchi, A., Fogli, P.G., Lovato, T., Peano, D., Iovino, D., Gualdi, S., Masina, S., Scoccimarro, E., Materia, S., Bellucci, A., et al. (2019). Global Mean Climate and Main Patterns of Variability in the CMCC-CM2 Coupled Model. J Adv Model Earth Syst *11*, 185–209. https://doi.org/10.1029/2018MS001369.

S2. Lovato, T., Peano, D., Butenschön, M., Materia, S., Iovino, D., Scoccimarro, E., Fogli, P.G., Cherchi, A., Bellucci, A., Gualdi, S., et al. (2022). CMIP6 Simulations With the CMCC Earth System Model (CMCC-ESM2). J Adv Model Earth Syst *14*,

e2021MS002814. https://doi.org/10.1029/2021MS002814.

S3. Döscher, R., Acosta, M., Alessandri, A., Anthoni, P., Arsouze, T., Bergman, T., Bernardello, R., Boussetta, S., Caron, L.-P., Carver, G., et al. (2022). The EC-Earth3 Earth system model for the Coupled Model Intercomparison Project 6. Geosci. Model Dev. *15*, 2973–3020. https://doi.org/10.5194/gmd-15-2973-2022.

S4. Müller, W.A., Jungclaus, J.H., Mauritsen, T., Baehr, J., Bittner, M., Budich, R., Bunzel, F., Esch, M., Ghosh, R., Haak, H., et al. (2018). A Higher‐resolution Version of the Max Planck Institute Earth System Model (MPI‐ESM1.2‐HR). J Adv Model Earth Syst *10*, 1383–1413. https://doi.org/10.1029/2017MS001217.

S5. Kawai, H., Yukimoto, S., Koshiro, T., Oshima, N., Tanaka, T., Yoshimura, H., and Nagasawa, R. (2019). Significant improvement of cloud representation in the global climate model MRI-ESM2. Geosci. Model Dev. *12*, 2875–2897. https://doi.org/10.5194/gmd-12-2875-2019.
